# Modified split bone block technique for reconstruction of the atrophic edentulous maxilla: a retrospective clinical study

**DOI:** 10.1186/s40729-026-00724-z

**Published:** 2026-10-17

**Authors:** Serhat Can, Sinem Büşra Kıraç Can, Gühan Dergin

**Affiliations:** 1 Oral and Maxillofacial Surgeon, Istanbul, Turkey; 2 Department of Oral and Maxillofacial Surgery, Marmara University Faculty of Dentistry, Istanbul, Turkey; 3 Dr. Serhat Can Private Clinic, Kadıköy-Istanbul, Turkey

**Keywords:** Modified split bone block technique, Atrophic maxilla, Alveolar ridge augmentation, Implant survival, Bone regeneration, Autologous bone grafting, Edentulous jaw

## Abstract

**Purpose:**

This study aimed to evaluate the modified split bone block technique in patients with an atrophic maxilla and to assess its clinical outcomes in terms of horizontal ridge width gain and implant outcomes.

**Methods:**

This retrospective clinical study included 18 patients who underwent reconstruction of the atrophic maxilla using the modified bilateral split bone block technique between January 2020 and April 2024. Preoperative and postoperative evaluations included clinical examinations and radiographic assessments to evaluate ridge width changes and implant outcomes after graft healing. The mean follow-up period after prosthetic loading was 18.6 ± 8.6 months (range, 12–44 months). Postoperative complications such as dehiscence, infection, and graft resorption were also recorded.

**Results:**

A total of 18 patients (10 females, 8 males; mean age, 54.22 ± 7.12 years) were included. The patient-level mean ridge width increased from **2.39 ± 0.45 mm at T0 to 7.27 ± 0.89 mm at T1**, with a mean horizontal ridge width gain of **4.88 ± 0.66 mm (95% CI**,** 4.55–5.21 mm; *p* < 0.001)**. Minor complications were observed in four patients, including transient paresthesia in three patients and limited wound dehiscence in one patient, which healed without graft loss. After a mean healing period of **4.5 ± 0.8 months**, 96 implants were placed, all of which demonstrated 100% survival at the 12-month follow-up, with no pain, infection, or mobility observed during clinical evaluation.

**Conclusions:**

The modified bilateral split bone block technique resulted in substantial horizontal ridge width gain and favorable short-term implant outcomes in this selected patient cohort. The technique may represent a clinically useful reconstructive option for appropriately selected patients with sufficient residual bone height.

**Supplementary Information:**

The online version contains supplementary material available at https://doi.org/10.1186/s40729-026-00724-z.

## Background

Reconstruction of the atrophic maxilla remains one of the most challenging procedures in implant dentistry [1, 2]. Implant rehabilitation in these patients requires careful planning and individualized treatment strategies [3]. Several treatment modalities have been proposed for the management of severe maxillary atrophy, including zygomatic and pterygoid implants, subperiosteal implants, iliac crest bone grafting, custom-designed titanium meshes, and autogenous bone block augmentation [4–8]. The choice of treatment depends on multiple factors, including the patient’s systemic condition, the degree of bone resorption, the morphology of the residual alveolar bone, and both the clinician’s experience and patient preferences [9, 10].

Autogenous bone grafting has been widely used for alveolar ridge reconstruction for many years and is supported by numerous studies [11]. Because of its osteogenic potential, biocompatibility, and ability to generate sufficient bone volume, it has long been considered the gold standard for alveolar ridge augmentation [12]. However, despite these advantages, its popularity has declined in recent years. Donor-site morbidity, longer treatment periods, and the need for multiple surgical procedures may delay implant placement and increase patient discomfort [13].

The split bone block technique involves dividing harvested ramus bone blocks into thin cortical plates and filling the interpositional space with autogenous particulate bone [14]. Modifications of this technique using a combination of autogenous and allogeneic particulate graft materials have also been described [15, 16]. Although evidence is available for partial ridge reconstruction, clinical evidence regarding this modified approach for extensive reconstruction of the severely atrophic edentulous maxilla remains limited. The aim of this study was to evaluate the clinical outcomes of the modified bilateral split bone block technique for reconstruction of the atrophic edentulous maxilla, with particular emphasis on horizontal ridge width gain, postoperative complications, and implant outcomes.

## Methods

### Study design and study sample

This retrospective study included patients treated between January 2020 and April 2024. This study was reported in accordance with the Strengthening the Reporting of Observational Studies in Epidemiology (STROBE) statement [17]. Prior to study, all participants were informed about the nature of the study and provided written informed consent. The study protocol was approved by the Marmara University Medical Faculty Ethics Committee (No: 09.2024.197, Date: 09/02/2024). The study was conducted in accordance with the principles of the Declaration of Helsinki. The records of patients who underwent reconstruction of the edentulous atrophic maxilla using the modified bilateral split bone block technique at the study clinic were reviewed according to predefined inclusion and exclusion criteria. A total of 23 patient records were initially screened; however, only 18 patients met all inclusion criteria and had complete follow-up data and were therefore included in the final analysis. The inclusion criteria were as follows: (1) Patients diagnosed with moderate to severe maxillary atrophy, (2) Presence of sufficient vertical bone height (≥ 8 mm) but limited horizontal bone width (≤ 4 mm) (3) availability of complete clinical and radiographic records before and after surgery, (4) Patients treated with the modified split bone block technique as the primary augmentation procedure, (5) ASA physical status I–II, (6) non-smokers. Exclusion criteria were as follows: (1) systemic diseases affecting bone metabolism (2) History of radiation therapy in the head and neck region (3) heavy smoking habits and (4) Current or recent bisphosphonate or corticosteroid therapy. (5) presence of active oral infections; (6) incomplete follow-up or missing clinical and radiographic documentation.

Preoperative and postoperative evaluations included clinical examinations and radiographic assessments of the augmented regions, while ridge width changes were assessed using intraoperative clinical measurements and implant outcomes were evaluated after graft healing.

All surgical procedures were performed by the same experienced oral and maxillofacial surgeon under standardized clinical conditions. Cone beam computed tomography (CBCT) and panoramic radiographs were used for treatment planning prior to surgery. Patients presented with complete edentulism or teeth with a poor periodontal prognosis. 18 patients with atrophic maxillae underwent autogenous bone harvesting from the bilateral mandibular ramus. Two bone blocks were obtained from the bilateral ramus of each patient and subsequently divided longitudinally to obtain four split bone blocks. In some cases, one of these four split bone blocks was further divided into smaller pieces according to the recipient-site anatomy; however, these secondary divisions were not counted as additional split bone blocks. Therefore, four split bone blocks per patient were included in the standardized block count. The split bone blocks were fixed to the anterior and premolar regions of the maxilla using 1.4-mm titanium screws. The number of fixation screws used per patient ranged from 8 to 13. Overall, 72 split bone blocks were used in 18 patients. After a healing period of 4 months, an average of four to six implants were placed in the maxilla per patient. Following an additional 3-month osseointegration period, fixed prostheses with metal-supported ceramic prostheses or zirconia-supported ceramic prostheses, or titanium-framed acrylic prostheses were fabricated. All patients were followed for a minimum of one year after prosthetic loading. The mean follow-up period after prosthetic loading was 18.6 ± 8.6 months (range, 12–44 months).

### Surgical protocol

All surgical procedures were performed under aseptic conditions by the same experienced oral and maxillofacial surgeon under local anesthesia and intravenous sedation or general anesthesia. A mid-crestal incision with posterior vertical releasing incisions was made to expose the maxillary alveolar ridge. After careful elevation of a full-thickness mucoperiosteal flap, the anterior, premolar, and molar regions of the maxilla were evaluated for augmentation.

For block graft harvesting, bilateral vestibular incisions were made in the mandibular ramus region, which was selected as the donor site because it provides dense cortical bone with relatively low donor-site morbidity. After flap elevation, autogenous particulate bone was gently collected from the ascending ramus using a disposable SafeScraper device (META, Reggio Emilia, Italy). Subsequently, piezosurgical instruments (Mectron S.p.A., Genoa, Italy) were used to perform corticotomies along the ridge, including mesial, distal, and basal cuts. Bilateral ramus bone blocks were then carefully harvested with osteotomes. Additional particulate bone was obtained from the harvested blocks using the SafeScraper.

After graft harvesting was completed, the ramus bone blocks were longitudinally split into thinner cortical plates using 0.2-mm-thick diamond separation discs with outer diameters of 8, 10, and 19 mm (Frank Dental GmbH, Gmund am Tegernsee, Germany). The resulting four split bone blocks were fixed to the anterior and premolar–molar regions of the maxilla with titanium mini-screws (Dentreal Bonefix, Turkey), with 8–13 fixation screws used per patient. Care was taken to maintain a minimum 5-mm gap between the split bone blocks and the residual alveolar bone. This space was densely packed with a mixture of autogenous particulate bone and a small amount of allograft material (approximately 1 cc per patient on average) (Maxxeus Dental Solvita).

**Fig. 1:**
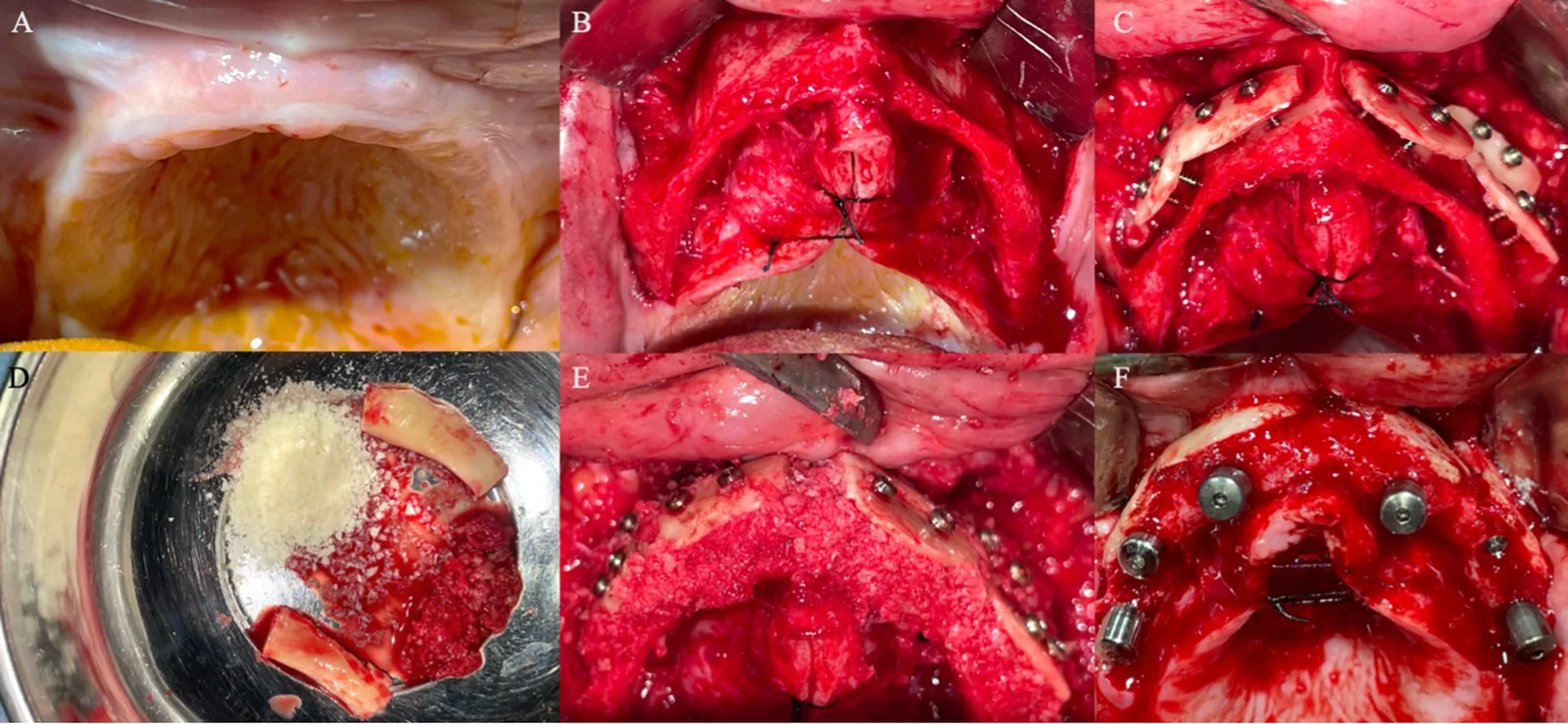
Intraoperative steps of the modified split bone block technique for maxillary ridge augmentation (Patient 1). (**A**) Atrophic maxillary ridge, (**B**) Flap elevation and site exposure, (**C**) Fixation of split bone plates with screws, (**D**) Harvested bone blocks and particulate graft, (**E**) Placement of particulate graft between plates, (**F**) Re-entry after healing and implant placement

**Fig. 2:**
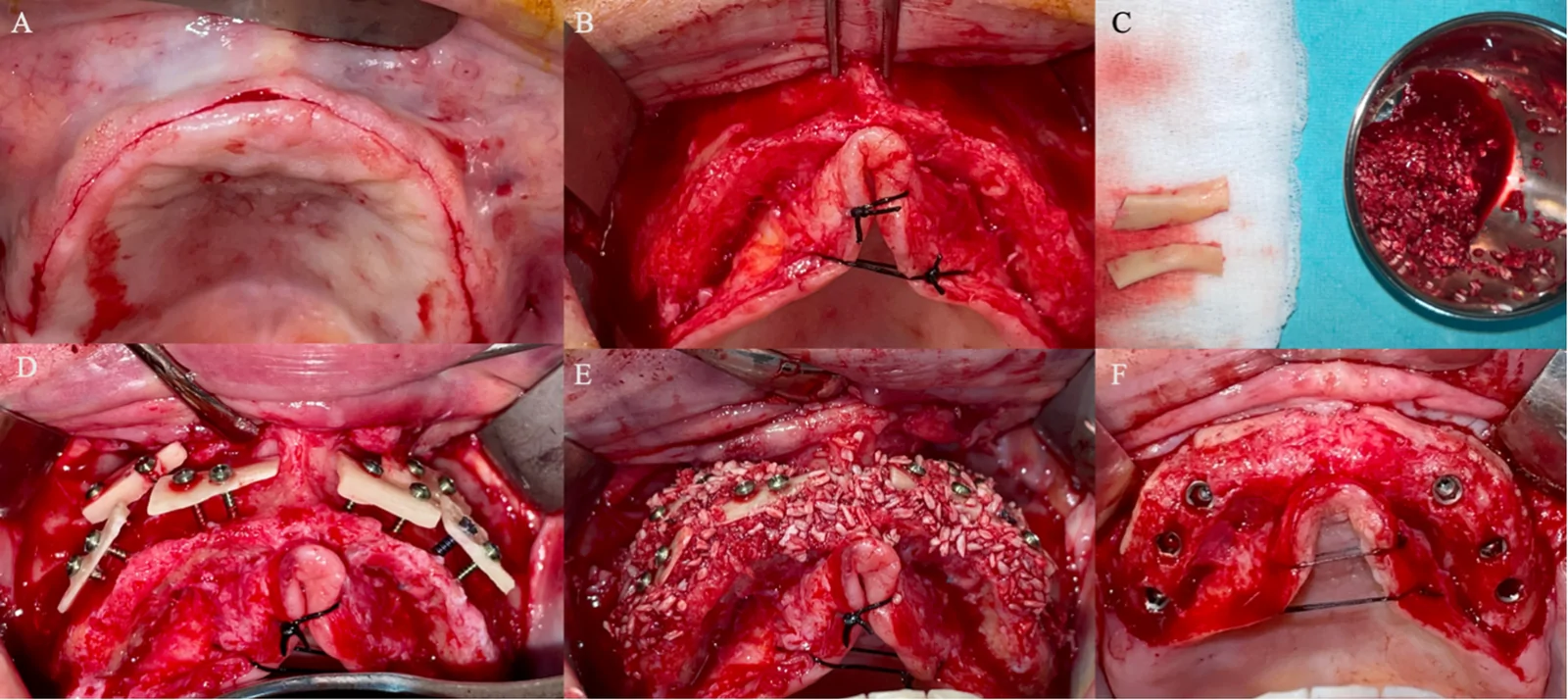
Intraoperative steps of the modified split bone block technique for reconstruction of the atrophic maxilla (Patient 2). (**A**) Crestal incision and flap design, (**B**) Flap elevation and exposure of the recipient site, (**C**) Harvested bone blocks and particulate graft material, (**D**) Fixation of split bone plates with screws, (**E**) Placement of particulate graft between the plates, (**F**) Re-entry after healing and implant placement

**Fig. 3:**
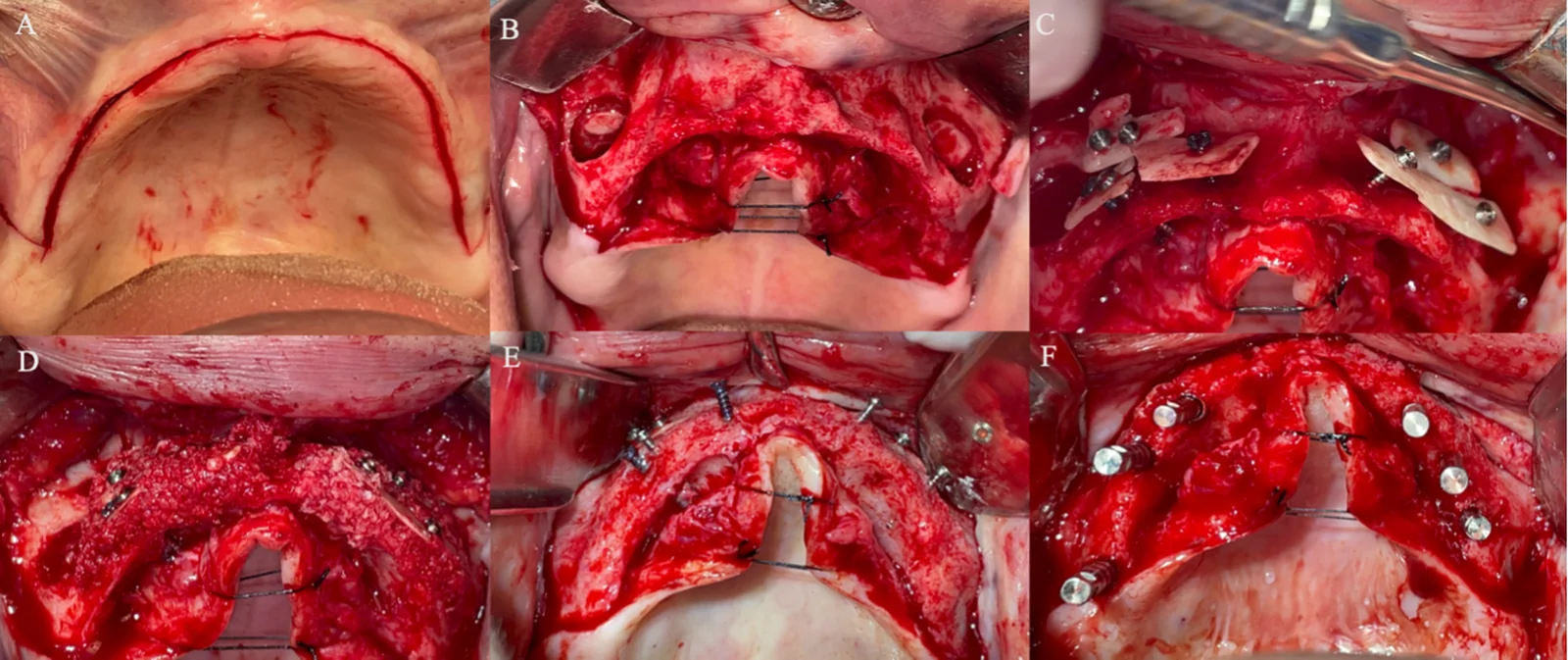
Intraoperative steps of the modified split bone block technique for reconstruction of the atrophic maxilla (Patient 3). (**A**) Crestal incision and flap design, (**B**) Flap elevation and exposure of the recipient site, (**C**) Fixation of split bone plates with screws, (**D**) Placement of particulate graft material, (**E**) Healed augmented ridge after the healing period, (**F**) Implant placement following re-entry

**Fig. 4:**
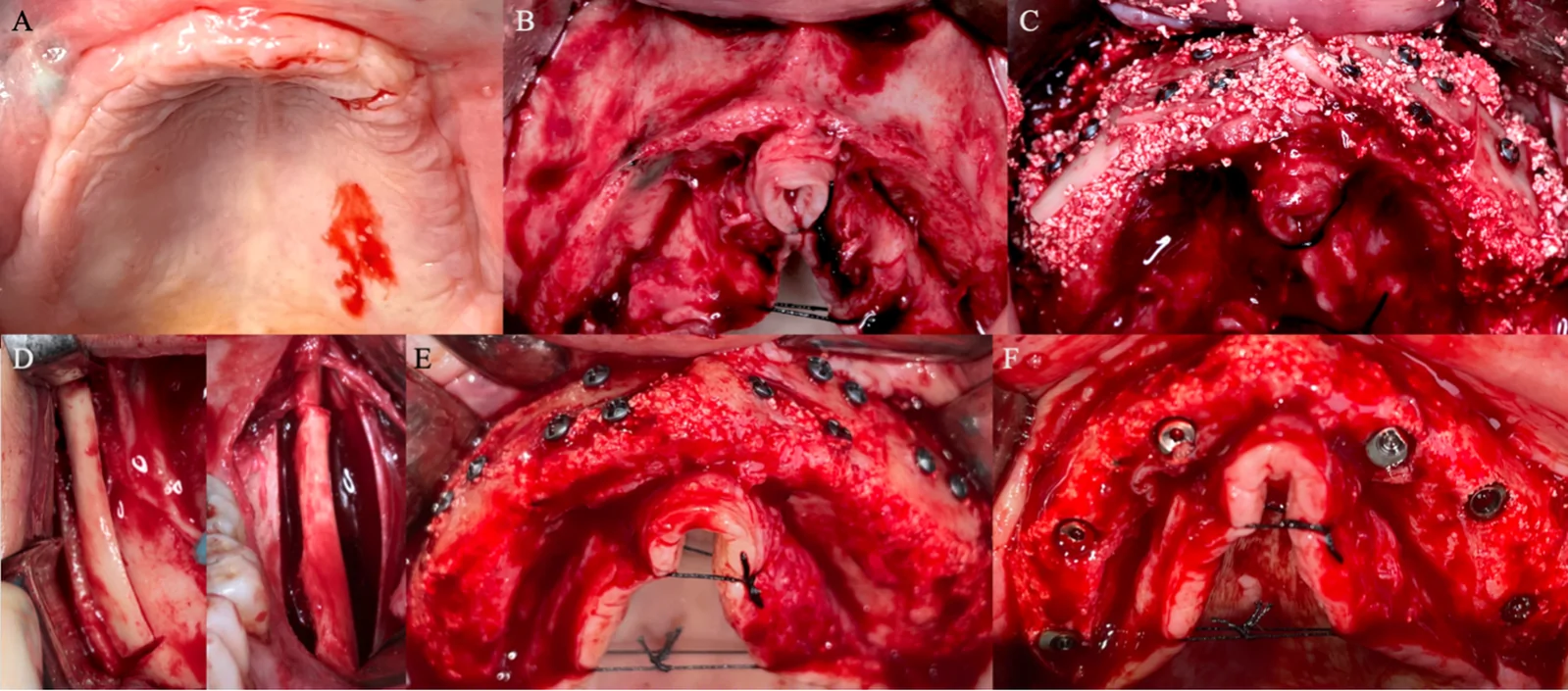
Intraoperative steps of the modified split bone block technique for reconstruction of the atrophic maxilla (Patient 4). (**A**) Atrophic maxillary ridge, (**B**) Flap elevation and exposure of the recipient site, (**C**) Fixation of split bone plates with screws and Placement of particulate graft between plates, (**D**) Harvested bone blocks, (**E**) Healed augmented ridge after the healing period, (**F**) Implant placement following re-entry

Periosteal-releasing incisions were made along the entire flap to ensure tension-free closure. After meticulous hemostasis, horizontal mattress and simple interrupted sutures were placed alternately, followed by a continuous locking suture for primary closure. All sutures were performed using 5 − 0 Monocryl (Doğsan, Turkey) with a 16-mm needle (Figs. 1, 2, 3 and 4).

Postoperative medications included oral amoxicillin/clavulanic acid (875/125 mg twice daily for 10 days), oral naproxen sodium (550 mg twice daily for 5 days), and a mouth rinse containing 0.12% chlorhexidine gluconate and 0.15% benzydamine hydrochloride three times daily for 10 days. oral paracetamol (500 mg, up to twice daily as needed) was additionally prescribed for pain control. This extended postoperative regimen reflected the clinical protocol used during the study period for patients undergoing extensive bilateral ramus bone harvesting and multi-region maxillary augmentation; similar postoperative antibiotic regimens have been reported in clinical studies involving maxillary bone augmentation [18, 19].

### Prosthetic protocol

Based on patient preferences, metal-supported ceramic prostheses, zirconia-supported ceramic prostheses, and titanium-framed acrylic prostheses were applied as early as 7 months after surgery. All prosthetic rehabilitation was performed using screw retainers.

### Follow-up visits and maintenance protocol

**Fig. 5:**
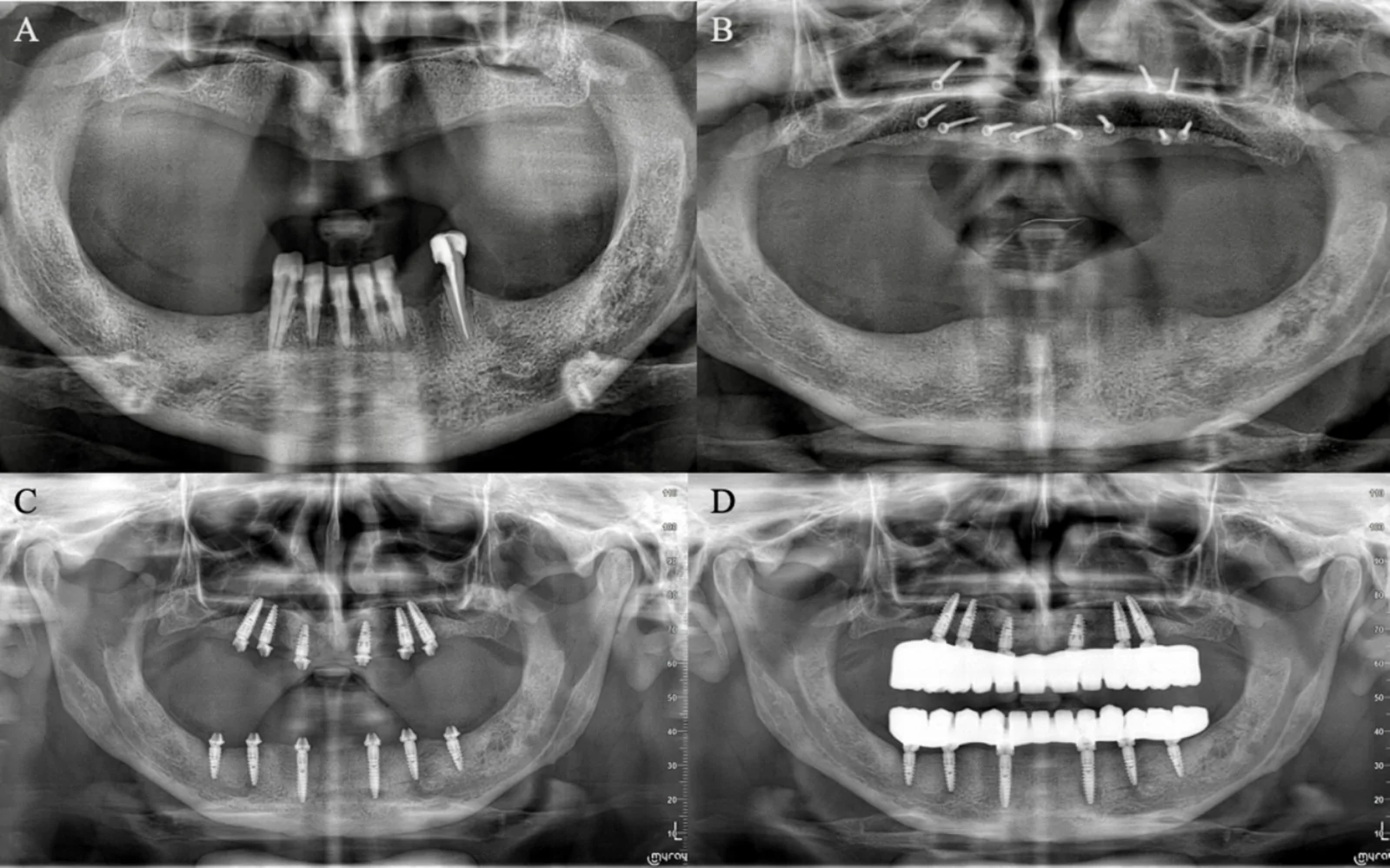
Panoramic radiographs of split bone block augmentation and implant rehabilitation (Patient 1). (**A**) Before bone augmentation, (**B**) Immediately after bone augmentation, (**C**) After implant placement, (**D**) 1 year after prosthetic rehabilitation

**Fig. 6:**
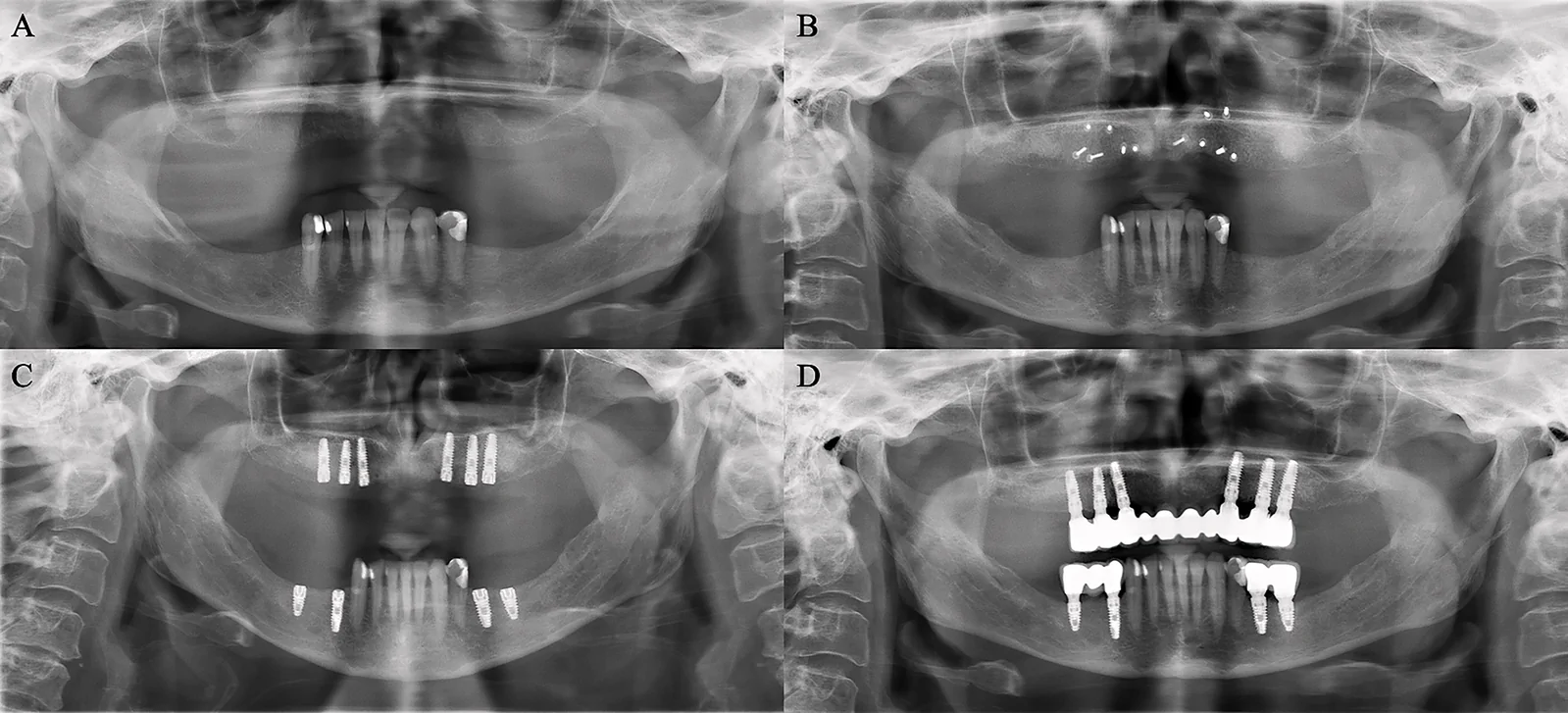
Panoramic radiographs of split bone block augmentation and implant rehabilitation (Patient 2). (**A**) Before bone augmentation, (**B**) Immediately after bone augmentation, (**C**) After implant placement, (**D**) 1 year after prosthetic rehabilitation

**Fig. 7:**
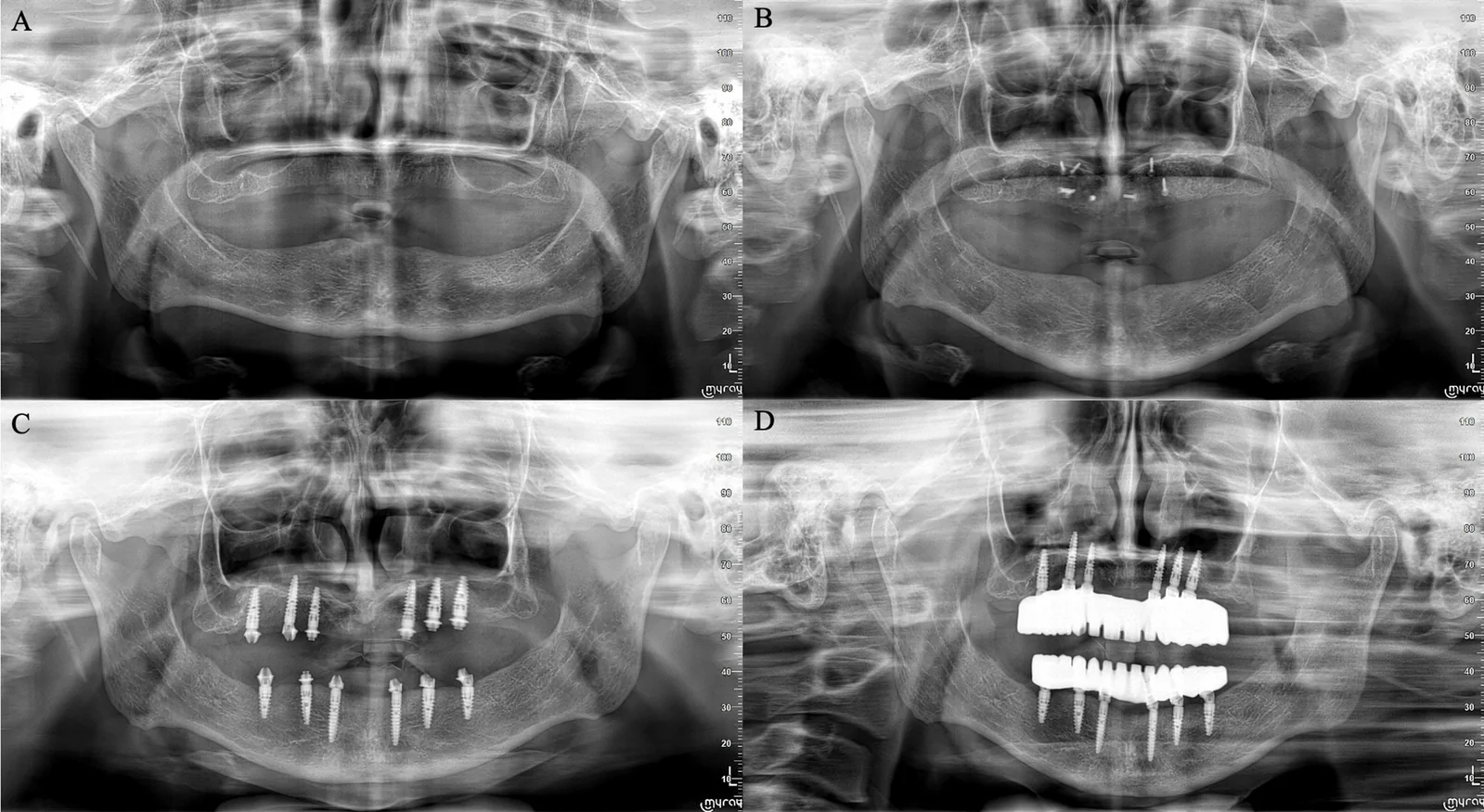
Panoramic radiographs of split bone block augmentation and implant rehabilitation (Patient 3). (**A**) Before bone augmentation, (**B**) Immediately after bone augmentation, (**C**) After implant placement, (**D**) 1 year after prosthetic rehabilitation

The authors instructed the patients to follow a strict protocol for the maintenance of oral hygiene after surgery. Follow-up visits were performed at 3 and 10 days after surgery, 2 and 4 months, and 1 year after prosthetic rehabilitation. Routine check-ups consisted of clinical evaluation, prophylaxis, and reinforcement of oral hygiene instructions (Figs. 5, 6 and 7).

### Data collection

Crestal ridge width was measured intraoperatively immediately after elevation of the mucoperiosteal flap during the initial surgery (T0) and reassessed during the re-entry procedure for fixation screw removal and implant placement (T1). Measurements were obtained at the alveolar crest using a calibrated periodontal probe and represented the horizontal buccopalatal ridge width, measured from the outer surface of the buccal cortical plate to the outer surface of the palatal cortical plate. Ridge width measurements were obtained from four predefined regions in each patient: the right and left anterior maxilla and the right and left premolar regions corresponding to the sites where the split bone blocks were fixed.

Cone-beam computed tomography (CBCT) scans were obtained preoperatively (T0) and before implant placement (T1) to evaluate the augmented region and ensure correct anatomical localization. However, due to scattering artifacts and the difficulty in clearly distinguishing the newly formed bone volume in postoperative images, radiographic measurements were not considered reliable for quantitative evaluation. Therefore, intraoperative measurements were used as the primary reference for assessing ridge width changes.

All measurements were performed using the same standardized protocol by the operating surgeon, who was not blinded to the treatment stage. For each patient, measurements obtained from the four standardized regions were averaged separately at T0 and T1 to obtain a single patient-level mean ridge width value for each time point. These patient-level mean values were used for the primary statistical analysis to avoid treating multiple measurements from the same patient as independent observations.

Clinically, wound healing, soft tissue closure, and postoperative complications such as dehiscence, infection, or graft exposure were recorded at each follow-up visit. After a healing period of 4 months, re-entry surgery was performed for fixation screw removal and implant placement. The newly formed bone was clinically assessed for quality, density, and stability during implant site preparation. Implant survival was defined as the implant remaining in situ and functional at the 12-month follow-up. Clinical implant outcomes were additionally assessed based on the absence of pain, infection, and mobility.

### Statistical analysis

All statistical analyses were conducted using IBM SPSS Statistics for Windows, Version 25.0 (IBM Corp., Armonk, NY, USA). Descriptive statistics were expressed as mean ± standard deviation (SD) for continuous variables and as frequencies and percentages for categorical variables.

For the primary outcome, the four regional ridge width measurements obtained from each patient were averaged separately at T0 and T1 to generate one patient-level mean ridge width value for each time point. The normality of the paired differences between the patient-level T0 and T1 mean values was assessed using the Shapiro–Wilk test. As the normality assumption was not violated, the patient-level mean ridge width values at T0 and T1 were compared using a paired-samples t-test. The mean change was reported with its standard deviation and 95% confidence interval (CI). Effect size was calculated as Cohen’s dz for paired observations. A two-sided p-value < 0.05 was considered statistically significant.

## Results

A total of 23 patient records were initially screened. Five patients were excluded from the final analysis: one because of incomplete data and four because of incomplete follow-up. Therefore, 18 patients met the eligibility criteria and were included in the final analysis in this retrospective study. The study population consisted of 8 males and 10 females, with a mean age of 54.2 ± 7.1 years. Ten patients were classified as ASA I and eight as ASA II. Each patient received four split bone blocks, resulting in a total of 72 split bone blocks used for ridge augmentation. The mean operative time was 227.6 ± 24.6 min. A total of 96 implants were placed in the augmented areas after a mean healing period of 4.5 ± 0.8 months (Table 1). The mean follow-up period after prosthetic loading was 18.6 ± 8.6 months (range, 12–44 months).

**Table 1:** Demographic and perioperative data

Variable		Mean ± SD (range), or *n* (%)
Sex	Male	8 (44.4)
	Female	10 (55.5)
Age, years		54.22 ± 7.12
ASA physical status	ASA-I	10 (55.5)
	ASA-II	8 (44.4)
Number of the split bone blocks		72
Implants placed in augmented region		96
Number of the donor sites		36
Operative time (minutes)		227.61 ± 24.62
Healing period after bone augmentation (months)		4.5 ± 0.8

Data are given in mean ± SD (standard deviation), numbers and percentages (%), unless otherwise stated

Descriptive ridge width measurements increased from T0 to T1 in all four predefined regions: from 2.46 ± 0.49 mm to 7.36 ± 0.90 mm in the right anterior region, from 2.35 ± 0.43 mm to 7.22 ± 0.99 mm in the right premolar region, from 2.37 ± 0.51 mm to 7.22 ± 0.89 mm in the left anterior region, and from 2.37 ± 0.54 mm to 7.28 ± 0.91 mm in the left premolar region. These regional measurements are presented descriptively, whereas the primary statistical analysis was performed using the patient-level mean ridge width.

**Fig. 8:**
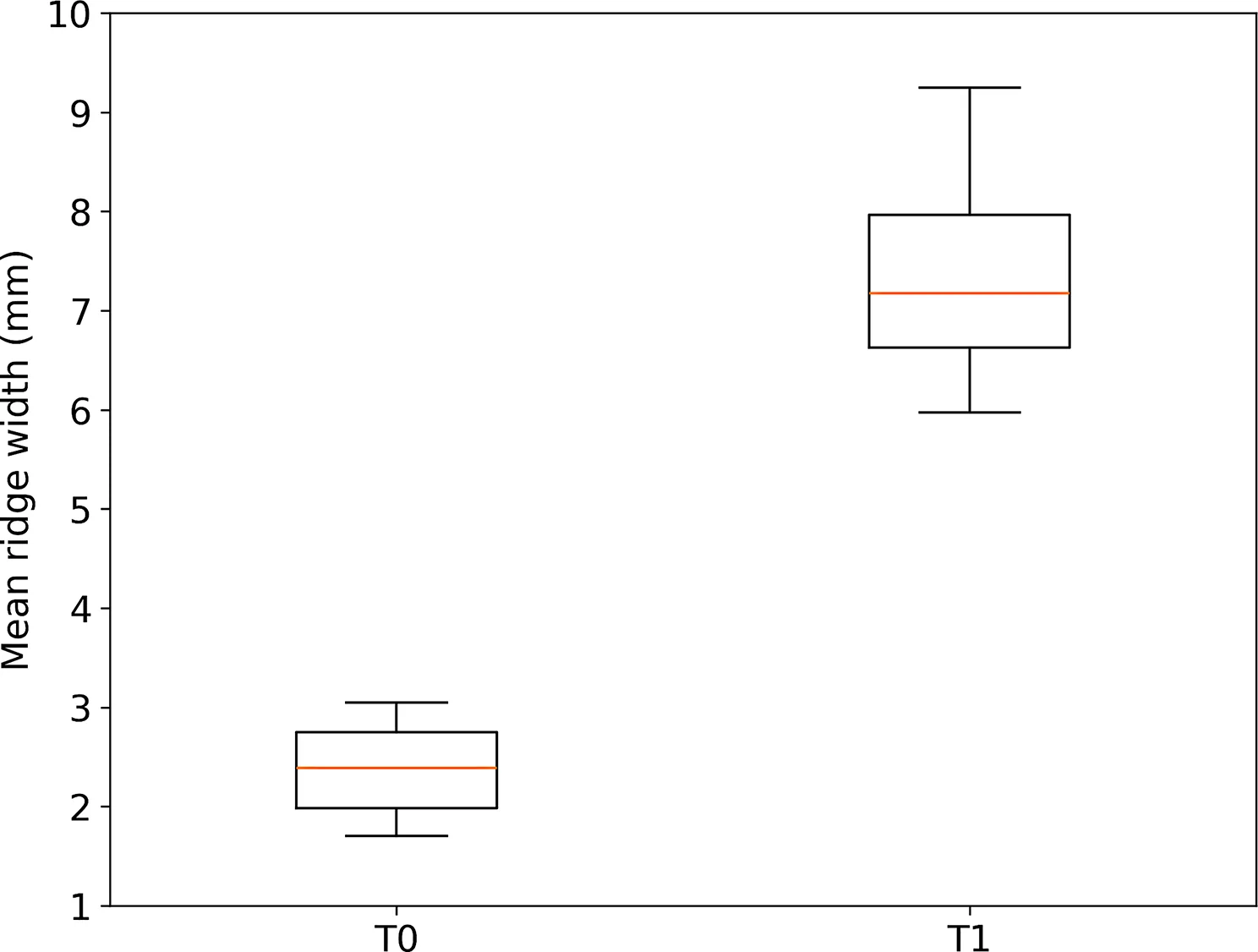
Distribution of patient-level mean alveolar ridge width at T0 and T1. Patient-level values were calculated by averaging measurements from the four predefined regions for each patient at each time point. T0, preoperative; T1, re-entry before implant placement

For each patient, the four regional measurements were averaged separately at T0 and T1 to obtain a single patient-level mean value for each time point. The patient-level mean ridge width increased from 2.39 ± 0.45 mm at T0 to 7.27 ± 0.89 mm at T1, corresponding to a mean horizontal ridge width gain of 4.88 ± 0.66 mm (95% CI, 4.55–5.21 mm). The distribution of the paired differences did not significantly deviate from normality (Shapiro–Wilk W = 0.898, *p* = 0.054). The difference between T0 and T1 was statistically significant (paired-samples t-test, t(17) = 31.19, *p* < 0.001), with a Cohen’s dz of 7.35 (Table 2; Fig. 8).

**Table 2:** Ridge width measurements and patient-level primary analysis

Measurement level / region	T0, mean ± SD (mm)	T1, mean ± SD (mm)	Mean change ± SD (mm)	95% CI	t (df)	*p*-value	Cohen’s dz
Regional descriptive measurements							
Right anterior	2.46 ± 0.49	7.36 ± 0.90	—	—	—	—	—
Right premolar	2.35 ± 0.43	7.22 ± 0.99	—	—	—	—	—
Left anterior	2.37 ± 0.51	7.22 ± 0.89	—	—	—	—	—
Left premolar	2.37 ± 0.54	7.28 ± 0.91	—	—	—	—	—
Patient-level primary analysis	**2.39 ± 0.45**	**7.27 ± 0.89**	**4.88 ± 0.66**	**4.55–5.21**	**31.19 (17)**	**< 0.001**	**7.35**

Data are presented as mean ± standard deviation (SD). Regional measurements are presented descriptively and were not analyzed as independent observations. For the primary analysis, the four regional measurements were averaged separately at T0 and T1 to obtain one patient-level mean ridge width value for each patient at each time point. Patient-level T0 and T1 values were compared using a paired-samples t-test. CI, confidence interval; T0, preoperative; T1, re-entry before implant placement

All patients completed the follow-up period without major complications. Minor complications were observed in four cases. In one patient, limited wound dehiscence with bone exposure occurred and healed uneventfully with conservative management without graft loss. In another patient, slight mobility of one split bone block was detected during implant placement; the block was stabilized with an additional fixation screw, and implant placement was completed as planned (Table 3).

**Table 3:** Results for the secondary outcomes

Secondary Outcomes		*n*(%)
Transient Paresthesia		3 (16.6)
Dehiscence		1 (5.55)
Infection		0 (0)
Implant loss		0 (0)
Intraoperative bone quality in augmented region (Lekholm&Zarb)	D1	0 (0)
	D2	6 (33.3)
	D3	10 (55.5)
	D4	2 (11.1)

Data are given in numbers and percentages (%), unless otherwise stated

**Fig. 9:**
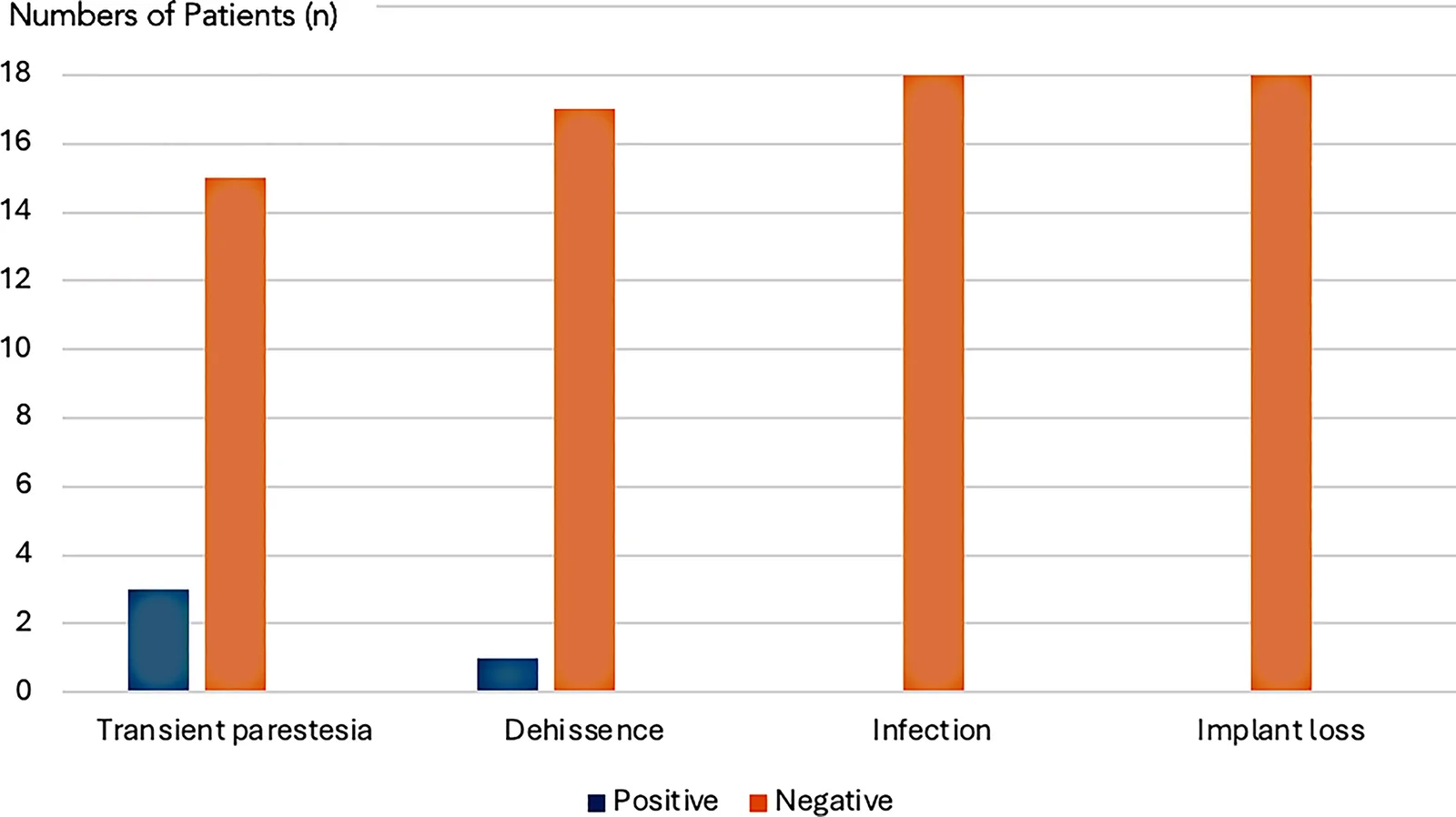
Distribution of postoperative complications. Bar chart showing the number of patients with positive and negative findings for transient paresthesia, wound dehiscence, infection, and implant loss

Transient unilateral paresthesia was observed in three patients and resolved completely within the one-year follow-up period. No cases of infection, graft failure, or significant graft resorption were recorded (Fig. 9) (Table 3).

Intraoperative assessment during implant site preparation indicated bone quality corresponding to Type II in six patients, Type III in ten patients, and Type IV in two patients. The maximum insertion torque was 70 Ncm and the minimum was 15 Ncm. All implants achieved primary stability at placement and demonstrated a survival rate of 100% at the 12-month follow-up. Clinical evaluation revealed no pain, infection, or implant mobility during this follow-up period (Table 3).

## Discussion

Reconstruction of the severely atrophic maxilla remains challenging, mainly due to the insufficient bone volume required for predictable implant placement [20]. The present study evaluated the clinical outcomes of the modified split bone block technique for reconstruction of the atrophic edentulous maxilla. The results demonstrated substantial horizontal bone gain (approximately 5 mm) together with high implant survival. A total of 96 implants were placed in the augmented areas, all of which remained functional during the follow-up period. In addition, the technique allowed reconstruction of multiple maxillary regions using split cortical plates harvested from bilateral ramus blocks. These findings support the clinical feasibility of the modified technique as a reconstructive option in appropriately selected patients with moderate to severe maxillary ridge deficiency.

Previous studies have reported comparable outcomes, indicating that split bone block augmentation can provide predictable horizontal bone regeneration when appropriate case selection and atraumatic surgical techniques are applied [21]. Autogenous bone grafts remain gold standard for alveolar ridge augmentation because of their biological compatibility and predictable integration [22]. Tolstunov et al. emphasized that stable fixation and proper soft-tissue management are critical factors for successful incorporation of autogenous grafts [22]. Similarly, Khoury and Hanser reported that the split bone block approach allows efficient use of cortical bone while maintaining favorable graft stability and vascularization [23]. In addition, several clinical studies have demonstrated that autogenous block grafting can achieve substantial horizontal ridge augmentation with relatively low complication rates when adequate residual bone is present for graft stabilization [24]. However, unlike the original fully autogenous split bone block technique, the modified protocol used in the present study combined autogenous cortical plates and particulate bone with a small amount of allogeneic particulate graft material. The addition of a small amount of allogeneic particulate material was intended to supplement the autogenous particulate bone because reconstruction of multiple maxillary regions required a relatively large interpositional graft volume. Obtaining the entire particulate graft volume from autogenous bone would have required more extensive bone scraping and prolonged the harvesting procedure. While the cortical plates and part of the particulate graft remained autogenous, the addition of allogeneic particulate material represents a relevant difference from the original biological concept. Therefore, the remodeling characteristics and long-term stability of this modified approach should not be assumed to be equivalent to those of the original technique. Furthermore, most of these studies mainly focused on partially edentulous ridges, whereas data regarding reconstruction of moderately to severely atrophic edentulous maxillae remain limited [24].

In the literature, iliac crest bone grafts are generally preferred for the reconstruction of extensive maxillary defects and advanced atrophy, where large graft volumes are required [10]. Iliac crest have been widely used for total maxillary augmentation; however, several studies have reported higher resorption rates and lower long-term implant survival compared with intraoral grafts [24]. In contrast, autogenous intraoral grafts are commonly preferred for localized augmentation procedures and have demonstrated more stable outcomes, with higher implant survival rates reported in long-term follow-up studies [25].

The posterior mandible, particularly the ramus region, represents an excellent donor site due to its dense cortical structure and favorable morphology for horizontal ridge augmentation [10, 24, 25]. Cortical block grafts harvested from this region provide high primary stability and reduced resorption compared with iliac grafts, which are characterized by a thinner cortex and a more porous cancellous component [10, 24–26]. In addition, the posterior mandible allows simultaneous harvesting of particulate bone using scraper devices, further enhancing grafting efficiency [10, 23].

In the present study, two block grafts harvested from the bilateral ramus were longitudinally divided to obtain four split cortical plates, which were sufficient to reconstruct the anterior and premolar regions of the maxilla. This approach allowed efficient utilization of intraoral donor bone and avoided the need for extraoral graft harvesting. From a biological perspective, the greater cortical density and membranous origin of mandibular bone may contribute to reduced graft resorption and improved stability compared with iliac crest grafts [10, 24–26].

Despite the favorable outcomes reported with autogenous augmentation, alternative graftless approaches have become increasingly popular in the management of the severely atrophic maxilla [25, 27]. Among these, zygomatic implants and digitally designed subperiosteal implants represent valuable treatment alternatives, particularly for patients who wish to avoid multiple surgical stages and prolonged edentulous periods. However, these treatments are technically demanding and require careful case selection and advanced surgical experience [28, 29]. Biological and prosthetic complications have been reported in the literature, including sinus-related complications, soft tissue dehiscence, implant exposure, and prosthetic maintenance challenges [29–31]. Similarly, modern additively manufactured subperiosteal implants have re-emerged as a promising option for severe maxillary atrophy, especially in patients who are not ideal candidates for conventional grafting [29]. Nevertheless, these implants also require individualized digital planning and careful soft tissue management, and implant exposure remains one of the most frequently reported complications [32–34]. In addition, both zygomatic and subperiosteal implants should generally be considered alternative or rescue options rather than the first choice in middle-aged patients with adequate donor bone, because failure of these treatments may severely limit or even prevent further implant-based rehabilitation [28, 35, 36]. In contrast, when sufficient donor bone is available, autogenous augmentation allows the placement of conventional implants and preserves future retreatment options and re-implantation possibilities.

In addition to autogenous block grafting, several augmentation techniques have been proposed for the reconstruction of severely resorbed maxillae [10, 25, 37]. Among these, customized titanium meshes produced through digital planning and additive manufacturing have gained increasing attention because they allow precise adaptation to complex defect morphologies and may facilitate guided bone regeneration in challenging cases [38]. However, clinical studies have reported complications such as mesh exposure, soft tissue dehiscence, and infection, which may compromise the regenerative outcome and require additional interventions. In addition, these digitally manufactured systems often require advanced planning workflows and may increase treatment costs [39]. As an alternative, commercially available allogeneic or xenogeneic bone blocks have also been introduced to eliminate donor-site morbidity associated with autogenous bone harvesting [16]. Although promising clinical results have been reported, concerns remain regarding their volume stability, and potential graft resorption compared with autogenous bone [40, 41]. Therefore, despite the development of these modern regenerative approaches, autogenous bone grafting continues to be considered a reliable option for ridge reconstruction when adequate donor bone is available.

This study has several limitations. The retrospective design and relatively small sample size may limit the generalizability of the findings. In addition, the follow-up period mainly reflects short- to mid-term outcomes, and long-term volumetric changes in the augmented bone were not evaluated; therefore, the long-term dimensional stability of the reconstructed ridge could not be determined. Ridge width measurements were performed by a single unblinded operating surgeon, and inter- and intra-examiner reliability were not assessed; therefore, potential measurement error cannot be excluded. In addition, CBCT measurements were not used for quantitative ridge width assessment because postoperative imaging artifacts limited reliable delineation of the newly formed bone; therefore, the primary outcome relied on intraoperative clinical measurements. The absence of a control group also prevents direct comparison with other augmentation techniques. Further prospective studies with larger cohorts and longer follow-up are needed to validate these findings and better define the indications of this technique in the era of digital and patient-specific reconstruction.

## Conclusions

Within the limitations of this retrospective study, the modified split bone block technique resulted in substantial horizontal ridge width gain and favorable short-term implant outcomes in this selected cohort of patients with moderate to severe maxillary atrophy. These findings support its clinical feasibility in appropriately selected patients; however, larger prospective studies with longer follow-up are needed to further evaluate the long-term stability and predictability of this modified approach.

## Supplementary Information

Below is the link to the electronic supplementary material.

Supplementary Material 1

## Data Availability

Data are available from the corresponding author on reasonable request.

## Abbreviations

CBCT: Cone beam computed tomography
T0: Preoperatively
T1: Before implant placement
SD: Standard deviation
