# Supplementary material for: Modified split bone block technique for reconstruction of the atrophic edentulous maxilla: a retrospective clinical study
Source: Int J Implant Dent. 2026 Oct 17;12:53. doi: 10.1186/s40729-026-00724-z (PMC13680584; doi:10.1186/s40729-026-00724-z)
Supplement: Supplementary file 1 [file 40729_2026_724_MOESM1_ESM.docx]

**STROBE Statement—Checklist of items that should be included in reports of cohort studies**

Manuscript: Modified Split Bone Block Technique for Reconstruction of the Atrophic Edentulous Maxilla: A Retrospective Clinical Study

| **Item** | **No.** | **Recommendation** | **Page No.** |
| --- | --- | --- | --- |
| Title and abstract | 1(a) | Indicate the study’s design with a commonly used term in the title or the abstract | 1–2 |
|  | 1(b) | Provide in the abstract an informative and balanced summary of what was done and what was found | 2 |
| Introduction — Background/rationale | 2 | Explain the scientific background and rationale for the investigation being reported | 3 |
| Introduction — Objectives | 3 | State specific objectives, including any prespecified hypotheses | 3 |
| Methods — Study design | 4 | Present key elements of study design early in the paper | 4 |
| Methods — Setting | 5 | Describe the setting, locations, and relevant dates, including periods of recruitment, exposure, follow-up, and data collection | 4–6 |
| Methods — Participants | 6(a) | Give the eligibility criteria, and the sources and methods of selection of participants. Describe methods of follow-up | 4–6 |
|  | 6(b) | For matched studies, give matching criteria and number of exposed and unexposed | N/A — not a matched study |
| Methods — Variables | 7 | Clearly define all outcomes, exposures, predictors, potential confounders, and effect modifiers. Give diagnostic criteria, if applicable | 4, 6–7 |
| Methods — Data sources/measurement | 8* | For each variable of interest, give sources of data and details of methods of assessment (measurement). Describe comparability of assessment methods if there is more than one group | 6–7 |
| Methods — Bias | 9 | Describe any efforts to address potential sources of bias | 4, 7 |
| Methods — Study size | 10 | Explain how the study size was arrived at | 4 |
| Methods — Quantitative variables | 11 | Explain how quantitative variables were handled in the analyses. If applicable, describe which groupings were chosen and why | 7-8 |
| Methods — Statistical methods | 12(a) | Describe all statistical methods, including those used to control for confounding | 7-8 |
|  | 12(b) | Describe any methods used to examine subgroups and interactions | N/A — no subgroup or interaction analyses |
|  | 12(c) | Explain how missing data were addressed | 4, 8 |
|  | 12(d) | If applicable, explain how loss to follow-up was addressed | 4–6, 8 |
|  | 12(e) | Describe any sensitivity analyses | N/A — no sensitivity analyses |
| Results — Participants | 13(a) | Report numbers of individuals at each stage of study—e.g., numbers potentially eligible, examined for eligibility, confirmed eligible, included in the study, completing follow-up, and analysed | 4, 8 |
|  | 13(b) | Give reasons for non-participation at each stage | 8 |
|  | 13(c) | Consider use of a flow diagram | Not used |
| Results — Descriptive data | 14(a) | Give characteristics of study participants (e.g., demographic, clinical, social) and information on exposures and potential confounders | 8; Table 1 |
|  | 14(b) | Indicate number of participants with missing data for each variable of interest | 4,8 |
|  | 14(c) | Summarise follow-up time (e.g., average and total amount) | 5, 8 |
| Results — Outcome data | 15* | Report numbers of outcome events or summary measures over time | 8–9; Tables 2–3 |
| Results — Main results | 16(a) | Give unadjusted estimates and, if applicable, confounder-adjusted estimates and their precision (e.g., 95% confidence interval). Make clear which confounders were adjusted for and why they were included | 8; Table 2 |
|  | 16(b) | Report category boundaries when continuous variables were categorized | 4 |
|  | 16(c) | If relevant, consider translating estimates of relative risk into absolute risk for a meaningful time period | N/A — no relative-risk estimates |
| Results — Other analyses | 17 | Report other analyses done—e.g., analyses of subgroups and interactions, and sensitivity analyses | N/A — no additional subgroup, interaction, or sensitivity analyses |
| Discussion — Key results | 18 | Summarise key results with reference to study objectives | 9 |
| Discussion — Limitations | 19 | Discuss limitations of the study, taking into account sources of potential bias or imprecision. Discuss both direction and magnitude of any potential bias | 12 |
| Discussion — Interpretation | 20 | Give a cautious overall interpretation of results considering objectives, limitations, multiplicity of analyses, results from similar studies, and other relevant evidence | 9–12 |
| Discussion — Generalisability | 21 | Discuss the generalisability (external validity) of the study results | 12 |
| Other information — Funding | 22 | Give the source of funding and the role of the funders for the present study and, if applicable, for the original study on which the present article is based | 13 |

* Give information separately for exposed and unexposed groups, if applicable.

N/A = not applicable.
